# Antimicrobial activity of the bioactive components of essential oils from Pakistani spices against *Salmonella* and other multi-drug resistant bacteria

**DOI:** 10.1186/1472-6882-13-265

**Published:** 2013-10-14

**Authors:** Rasheeha Naveed, Iftikhar Hussain, Abdul Tawab, Muhammad Tariq, Moazur Rahman, Sohail Hameed, M Shahid Mahmood, Abu Baker Siddique, Mazhar Iqbal

**Affiliations:** 1 Institute of Microbiology, University of Agriculture, Faisalabad 38040, Pakistan; 2 Health Biotechnology Division, National Institute for Biotechnology and Genetic Engineering (NIBGE), Faisalabad 38000, Pakistan; 3 Department of Microbiology, Government College University, Faisalabad, Pakistan

**Keywords:** Essential oils, Multidrug resistant, Minimum inhibitory concentration, GC/MS, TLC-bioautography

## Abstract

**Background:**

The main objective of this study was the phytochemical characterization of four indigenous essential oils obtained from spices and their antibacterial activities against the multidrug resistant clinical and soil isolates prevalent in Pakistan, and ATCC reference strains.

**Methods:**

Chemical composition of essential oils from four Pakistani spices cumin (*Cuminum cyminum*), cinnamon (*Cinnamomum verum*), cardamom (*Amomum subulatum*) and clove (*Syzygium aromaticum*) were analyzed on GC/MS. Their antibacterial activities were investigated by minimum inhibitory concentration (MIC) and Thin-Layer Chromatography-Bioautographic (TLC-Bioautographic) assays against pathogenic strains *Salmonella typhi* (D1 Vi-positive), *Salmonella typhi* (G7 Vi-negative), *Salmonella paratyphi* A, *Escherichia coli* (SS1), *Staphylococcus aureus*, *Pseudomonas fluorescens* and *Bacillus licheniformis* (ATCC 14580). The data were statistically analyzed by using Analysis of Variance (ANOVA) and Least Significant Difference (LSD) method to find out significant relationship of essential oils biological activities at *p* <0.05.

**Results:**

Among all the tested essential oils, oil from the bark of *C. verum* showed best antibacterial activities against all selected bacterial strains in the MIC assay, especially with 2.9 mg/ml concentration against *S. typhi* G7 Vi-negative and *P. fluorescens* strains. TLC-bioautography confirmed the presence of biologically active anti-microbial components in all tested essential oils. *P. fluorescens* was found susceptible to *C. verum* essential oil while *E*. *coli* SS1 and *S. aureus* were resistant to *C. verum* and *A. subulatum* essential oils, respectively, as determined in bioautography assay. The GC/MS analysis revealed that essential oils of *C. cyminum*, *C. verum*, *A. subulatum*, and *S. aromaticum* contain 17.2% cuminaldehyde, 4.3% *t*-cinnamaldehyde, 5.2% eucalyptol and 0.73% eugenol, respectively.

**Conclusions:**

Most of the essential oils included in this study possessed good antibacterial activities against selected multi drug resistant clinical and soil bacterial strains. Cinnamaldehyde was identified as the most active antimicrobial component present in the cinnamon essential oil which acted as a strong inhibitory agent in MIC assay against the tested bacteria. The results indicate that essential oils from Pakistani spices can be pursued against multidrug resistant bacteria.

## Background

Resistance of pathogens to antimicrobial compounds has lethal effects as the development of drug resistance outpaces the development of new drugs. Infectious diseases, a leading cause of untimely death worldwide, have become a global concern. The clinical effectiveness of many existing antibiotics is being threatened by rapid emergence of multidrug resistant pathogens [1]. Many infectious diseases have been identified to be treated with herbal products throughout the history of mankind [2]. Natural products provide enormous opportunities for the development of new drugs, especially antimicrobials, which can have therapeutic potential to treat infectious diseases [3]. There is a continuous need to discover new antimicrobial compounds with suitable chemical structures and novel mode of actions against pathogens. Antimicrobial compounds of plant origin have an enormous therapeutic potential to treat many infectious diseases [3].

*Salmonella enterica* serovars *typhi*, *paratyphi* A & B, *typhimurium* and *enteritidis* represent sufficiently large disease burden to animal kingdom. Among these serovars, *S. typhi* (*S*. *typhi*) and *S. paratyphi* (*S*. *paratyphi*) are restricted to humans only and cause enteric fever commonly known as typhoid fever or simply typhoid. According to the global estimates conducted in 2000, around 27 million cases of typhoid fever with 216,000 deaths were reported to occur annually and more than 90% of these morbidities and mortalities occurred only in Asia. Enteric fever is highly prevalent in south Asia, Southeast Asia and the Indian Subcontinent [4]. Unfortunately, typhoid is proved to be the 4^th^ major cause of deaths in Pakistan [5]. Moreover, *S*. *typhi* and *S*. *paratyphi* have developed the resistance against first line of antibiotics [6]. In Pakistan, numbers of multidrug resistant (MDR) bacterial strains, belonging to *Salmonella spp*., have been isolated from typhoid patients’ blood [7-9]. Similarly, high frequency of Extended-Spectrum *ß* Lactamase (ESBL) producing organism belonging to *Enterobacter spp*. (50%), *E*. *coli* (41%), *Klebsiella*. *pneumonia* (36%) and nosocomial isolates (52%) have been reported from Pakistan [10]. About 22.3% population is living below the poverty line [11] and this low income group who cannot afford the second line expensive drugs is especially vulnerable to drug resistant pathogens. Moreover, certain *Salmonella* strains have developed resistance even against newer agents like azithromycin and the development of significant resistance against the current generation antibacterial agents can be envisioned in near future [12,13]. Therefore, some affordable and effective strategies are required to address these life threatening bacterial infections.

Essential oils have been known for centuries for their biological activities and have been widely evaluated against various biological targets [14] such as bactericidal [15], virucidal [16], fungicidal [17], antiparasitic [18], insecticidal [19], anticancer agents [20-22], cholesterol lowering agents [23], cosmetics [24] and other pharmaceutical applications [25]. These antimicrobial activities of essential oils have been resulted after screening of a wide range of plant species [15]. Essential oils can be a valuable source to explore their antibacterial properties against multidrug resistant human pathogens. The essential oils have already proved to exert strong synergistic effects when used in combination with less effective antibiotics [26]. The ethanol extract of *Turnera ulmifolia* when combined with gentamicin and kanamycin, has exerted a dramatic effect on the inhibition of multidrug resistant *S aureus*–MRSA strain [27]. In fact, essential oils extracted from edible plants are generally not harmful and thus are potential source of food additives [14,18].

Mostly plant derived essential oils consist of chemical components such as terpenoids including monoterpenes, sesquiterpenes and their oxygenated derivatives. These compounds have the ability to easily diffuse across cell membrane to induce biological reactions [28]. Chemical analysis has shown that the composition of essential oils from different plant species varies significantly. Even within the same species, plants belonging to different geographical sources, the composition of essential oils can differ reasonably [29]. Clove buds essential oil induced antimicrobial activity due to high level of eugenol and eucalyptol components [30]. Eugenol (4-allyl-2-methoxyphenol), in addition to clove also present in cinnamon oil is active against fungi, viruses and many pathogenic bacteria such as *E*. *coli*, *Listeria monocytogenes*, *Campylobacter jejuni*, *S enterica*, *S aureus*, *Lactobacillus sakei*, and *Helicobacter pylori*[31-33]. Cinnamon derived essential oil has been used to treat many diseases because of potential antimicrobial activity of its major component, cinnamaldehyde [34]. Essential oil of cardamom has been used in traditional medicine and its chemical composition showed that eucalyptol was the major responsible constituent for antimicrobial activity [35]. Cuminaldehyde is one of the major flavoring components of cumin essential oil that can induce different biological activities [36].

The present study was aimed to determine the chemical compositions and antibacterial properties of essential oils from common spices from Pakistan against different bacterial strains such as *S*. *typhi* (D1 Vi-positive), *S*. *typhi* (G7 Vi-negative), *S*. *paratyphi* A, *E*. *coli* (SS1), *S*. *aureus*, *P*. *fluorescens* (soil isolate) and *B*. *licheniformis* (ATCC 14580). The first five strains are the clinical isolates and have been identified as multidrug resistant bacteria. The selected bacterial strains are wide spread and cause serious problems due to their pathogenicities and high levels of drug resistance [7,37-40].

## Methods

### Essential oils

Dry mass of 1000 g of seeds of *C*. *cyminum* and *A*. *subulatum*, bark of *C*. *verum* and buds of *S*. *aromaticum* were subjected to distillation in hydro-distillation unit for 4–6 hours, as described previously (23). The essential oils obtained were dried over anhydrous sodium sulphate, filtered and stored at 4°C in sealed glass vials. Before extraction, the spices were identified and authenticated by Dr. Mansoor Hameed, Taxonomist of Department of Botany, University of Agriculture, Faisalabad-Pakistan. The specimens were further confirmed by comparing with authenticated samples. Voucher specimens of *C cyminum* (No. 7140/10.05.44), *A. subulatum* (No. 7090/20.05.34), *C. verum* (No. 7102/26.10.39) and *S. aromaticum* (No. 8023/30.06.45) were deposited in the Herbarium of University of Agriculture, Faisalabad-Pakistan. Standards, chemicals and HPLC grade organic solvents were obtained from Sigma Aldrich Fluka, Fisher and Merck.

### Test organisms

Bacterial strains used in this study were *S. enterica* serovar *typhi* (D1 Vi-positive and G7 Vi-negative strains), *S. enterica* serovar *paratyphi* A, *E. coli* (SS1), *S. aureus*, *B. licheniformis* (ATCC 14580), and *P. fluorescens*. *Salmonella* strains were isolated from the typhoid patients’ blood [37], and *E*. *coli* was isolated from wound infection [38]. *S*. *aureus* was also a clinical isolate (patient urine) while *P*. *fluorescens* was isolated from soil rhizospheres (un-published data). *S*. *typhi* D1 strain produce biofilm or Vi-polysaccharide around it and hence termed as Vi-positive, while *S*. *typhi* G7 does not produce Vi-polysaccharide and named as Vi-negative strain [41]. Among tested strains, MDR characteristic was present in three strains of *Salmonella spp*. [7], *E*. *coli*[38] and *S*. *aureus* (un-published data). These microorganisms were obtained from Health Biotechnology Division, National Institute for Biotechnology and Genetic Engineering (NIBGE), Faisalabad, Pakistan. These bacteria were maintained on LB agar medium (MP Biomedicals, France) at 4°C and cultured in LB broth at 37°C.

### Antibacterial assays

#### Minimum inhibitory concentrations (MIC)

The MIC values of essential oils were evaluated using broth microdilution assay in sterile 96-well microtiter plates in triplicate [36]. In each well, 60 μl of LB broth was added. The 60 μl of essential oil (500 mg/ml) was pipetted into the wells in the first column of the plate and two- fold serial dilution was prepared with LB broth. Then 60 μl of the overnight grown bacterial culture was added in each well. Microtiter plates were then incubated at 37°C for 24 hours. After incubation, wells were examined for microbial growth. Growth was determined by the turbidity of the culture media in the wells. Concentration of the first well without turbidity was considered as minimum inhibitory concentration. The inhibition of bacterial growth was determined by measuring absorbance at 600 nm with an ELISA reader. Amoxicillin (30 μg/ml) and DMSO (Dimethyl Sulfoxide, MP Biomedicals, France) were used as positive and negative control, respectively.

#### TLC − bioautography

Each of essential oil samples and reference standards were dissolved in ethyl acetate (10 μl/mL) and 8 μl of each from oils and standards were applied on to the two silica coated TLC plates (20 × 20 cm TLC silica gel 60-F_254_ aluminium sheet, Merck, Germany) by using capillary pipettes. Out of these, one TLC plate was marked for reference TLC chromatogram, while other was used for bioautography. Each standard was applied next to the spot of its essential oil on TLC chromatographic plate. TLC was optimized for these oils using *n*-hexane and ethyl acetate (9:1) at room temperature. After drying, TLC plates were examined under UV light (254 nm) and followed by dipping in alcoholic vanillin sulfuric acid reagent (prepared by dissolving, ethanol 95 ml, vanillin 6 g, and concentrated sulfuric acid 1.5 ml). These plates were heated using heat gun for 1 min to visualize the separated compounds which were detected on the basis of their *R*_f_ values visually and by the color of the spots generated [34].

The TLC plate used for bioautography was placed over LB agar containing 2 μl of overnight culture grown in LB broth at 37°C and 300 μl of aqueous solution of 3- (4, 5-dimethyl thiazol-2-yl)-2, 5-diphenyltetrazolium bromide (MTT, 0.05 g/30 ml). The treated plates were incubated at 37°C for 24 hours. The zones of inhibition were visualized as pale spots against a dark blue background and measured in mm unit. The inhibitory zones observed were then correlated with the spots seen on the TLC plate under UV light, which were saved as reference TLC plates [42]. The tests were performed in triplicates.

#### GC/MS analysis

The separation and identification of volatile components of essential oils were carried out by GC/MS (Trace GC Ultra coupled with ion trap Polaris Q mass spectrometer made by Thermo) [43]. The capillary column used was TR-5MS (30 m × 0.25 mm × 0.25 μm). The GC conditions were programmed as the injection temperature 230°C, with oven temperature initially set at 100°C for 2 min, then ramping @ 8°C/ min to 270°C, stayed for 5 min at this temperature. The injection mode was split @ (50:50), injection volume was 0.2 μl and carrier gas used was Helium (99.999%) at constant flow rate of 1 ml/min. The MS transfer line temperature was set at 260°C, with EI ionization mode at 70 eV ionization potential. The ion source temperature was 200°C and the analysis mass range was 50–300 m/z. Samples (300 μl) were run in *n*-hexane (Lab-scan, Thailand) with a dilution of 0.1 mg/ml. Compounds were identified by matching their mass spectra fragmentation pattern and retention time with standard reference compounds and comparing the MS results with NIST (National Institute of Standards and Technology) library stored in GC/MS database for confirmation.

### Statistical analysis

All the experimental results were performed in triplicate and the results were expressed as means ± SE and. The data were statistically analyzed using ANOVA and significant relationship at *p* < 0.05 with LSD method by using Minitab Release 15 software program.

## Results

The hydro-distillation process gave varied yields of essential oils from seeds of selected spices. *C*. *cyminum* gave the maximum yield (2.2%), followed by *S*. *aromaticum* (0.15%), *C*. *verum* (0.1%) and *A*. *subulatum* (0.1%). To investigate the antibacterial activities of essential oils, all bacterial strains were challenged with their different concentrations and the MIC was calculated by broth micro-dilution assay. Majority of essential oils exhibited antibacterial activities against the selected set of microorganisms (Table 1). The essential oil of *C*. *cyminum* produced significantly variable MIC values against the selected bacteria. It gave the better MIC value (3.4 mg/ml for each) against *S*. *typhi* D1 and *E*. *coli* SS1 strains but fairly lower activities against *S*. *paratyphi*, *P*. *fluorescens* and *S*. *aureus* strains. On the other hand, essential oil of *C*. *verum* gave the overall excellent activities against all the selected bacterial strains. Its MIC values ranged from 2.9 to 4.8 mg/ml. Essential oil of *A*. *subulatum* showed highest activities against *E*. *coli* SS1 strain demonstrating the best MIC value (2.83 mg/ml) among all the tested essential oils, while it also showed activity against other organisms. Similarly, essential oil of *S*. *aromaticum* exhibited significant inhibitory effect against all tested strains and gave better MIC values against three *Salmonella* strains as compared to the oil of *C*. *cyminum*. Amoxicillin was used as a positive control and all tested bacteria showed resistant against this antibiotic at concentration of 30 μg/ml in broth microdilution assay except *B*. *licheniformis* (Table 1, see footnote).

**Table 1:** MICs of essential oils against bacteria by micro broth dilution assay

**Bacterial strains**^**b**^		**Essential oils**^**a**^		
	***C. cyminum***	***C. verum***	***A. subulatum***	***S. aromaticum***
*S. typhi* D1	3.4 ± 0.87^*^	3.8 ± 0.96^*^	6.6 ± 2.4^*^	5.4 ± 1.08^*^
*S. typhi* G7	6.1 ± 2.3	2.9 ± 0.11	3.7 ± 0.94	3.26 ± 0.05
*S. paratyphi* A	14 ± 1.7^*^	3.8 ± 0.96	4.1 ± 0.94	4.3 ± 1.08
*E. coli* SS1	3.4 ± 0.87^*^	3.8 ± 0.96^*^	2.83 ± 0.11^*^	5.4 ± 1.08^*^
*S. aureus*	29.7 ± 1.7^*^	4.8 ± 0.96	9.4 ± 1.86	5.4 ± 1.08
*B. licheniformis*	4.3 ± 0.87	3.8 ± 0.96	4.7 ± 0.94	7.6 ± 2.8^*^
*P. fluorescens*	12.2 ± 1.7^*^	2.9 ± 0.12	7.5 ± 1.8	8.6 ± 2.1

^a^MIC are presented as mean of triplicate values ± SE in mg/ml; ^b^all tested bacteria showed resistant against amoxicillin (30 μg/ml) in MIC assay except *B. licheniformis*; ^*^significant values at *p* < 0.05.

**Figure 1:**
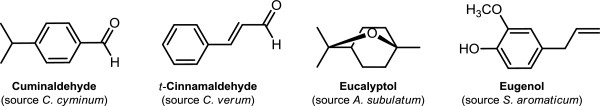
Structures of the active components from tested essential oils.

To further explore the antibacterial activities of the components of the essential oils, TLC-bioautography was used. TLC of each essential oil was performed on silica coated TLC plates in duplicate manner. The control TLC plates were treated with alcoholic-vanillin sulfuric acid reagent to spot the components while the second plates were used for bioautographic assay. The control TLC plate of essential oil from *C*. *cyminum* showed various bands at different *R*_f_ values but the main component was identified as cuminaldehyde (*R*_f_ = 0.55) when compared with its reference standard. The essential oils from *S*. *aromaticum*, *A*. *subulatum* and *C*. *verum* mainly consisted of eugenol (*R*_f_ = 0.22), eucalyptol (*R*_f_ = 0.52), and *t*-cinnamonaldehyde (*R*_f_ = 0.45), respectively (Figure 1). The *R*_f_ values of these bands were correlated by parallel running of authentic standards of these chemicals on TLC plates (Table 2). During this TLC-bioautography assay, antibacterial activities of the essential oils as well as their reference standards were expressed as diameter (mm) of the inhibition zones. This assay was conducted against the subjected microbial cultures i.e. *S*. *typhi* D1 Vi-positive, *S*. *typhi* G7 Vi-negative, *S*. *paratyphi* A, *E*. *coli* SS1, *S*. *aureus*, *B*. *licheniformis* ATCC 14580, and *P*. *fluorescens* (Table 2). From the oil of *C*. *cyminum*, cuminaldehyde and its standard showed good antibacterial activities against multidrug resistance strains from *Salmonella spp*.: *S*. *typhi* D1 Vi-positive (20 mm), *S*. *typhi* G7 Vi-negative (20.1 mm) and *S*. *paratyphi* A (24.5 mm). This was followed by moderate activities against *E*. *coli*, *S*. *aureus*, *and B*. *licheniformis* strains. However, neither *C*. *cyminum* cuminaldehyde nor its reference standard was able to show any antimicrobial activities against *P*. *fluorescens*.

**Table 2:** Efficacy of main components and reference standards of indigenous essential oils against tested bacteria using TLC-bioautography

**Bacterial strains**	**Essential oils**^**a**^							
	***C. cyminum***		***C. verum***		***A. subulatum***		***S. aromaticum***	
	**Cumin aldehyde *R***_**f**_^**b,c **^**0.55**	**Cumin aldehyde standard**	**Cinnam aldehyde *R***_**f**_^**b,c **^**0.45**	**Cinnam aldehyde standard**	**Eucalyptol *R***_**f**_^**b,c **^**0.52**	**Eucalyptol standard**	**Eugenol *R***_**f**_^**b,c **^**0.22**	**Eugenol standard**
***S. typhi *D1**	20.0 ± 0.57^d^	17.0 ± 0.57	24.6 ± 0.33^*^	24.0 ± 0.57^*^	25.0 ± 0.57^*^	22.3 ± 0.33	22.0 ± 0.57	24.6 ± 0.66^*^
***S. typhi *G7**	20.1 ± 0.60	18.5 ± 0.86	26.5 ± 0.28^*^	25.6 ± 0.66^*^	25.5 ± 0.28^*^	23.0 ± 0.57	21.5 ± 0.28	21.0 ± 0.57
***S. para typhi *A**	24.5 ± 0.28	22.0 ± 0.1	25.5 ± 0.28	22.3 ± 0.33	24.6 ± 0.33	23.0 ± 0.57	20.0 ± 0.57	22.3 ± 0.88^*^
***E. coli *SS1**	9.33 ± 0.33	8.66 ± 0.33	R^e^	R^e^	9.33 ± 0.33	11.3 ± 0.66	29 ± 0.57	31.6 ± 0.88^*^
***S. aureus***	12.0 ± 0.86	11.8 ± 0.60	23.5 ± 0.28^*^	23.16 ± 0.60^*^	R^e^	R^e^	22.6 ± 0.88^*^	25.3 ± 0.66^*^
***B. licheni formis***	15.6 ± 2.18	18.6 ± 2.66	32.6 ± 1.20^*^	31.6 ± 0.88^*^	20.3 ± 0.88	19.6 ± 0.88	16.3 ± 0.33	19.3 ± 0.33
***P. fluores cens***	R^e^	R^e^	15 ± 0.57	17.6 ± 0.33^*^	R^e^	R^e^	R^e^	R^e^

^a^diameter zone of growth inhibition were presented as mean ± S.E. in mm; ^b^retardation factor; ^c^mobile phase: *n*-hexane, ethyl acetate (9:1 v/v); ^d^each value is presented as mean of three replicate; ^e^resistant; ^*^showed significant (*p* <0.05) values.

The main constituent of essential oils from *C*. *verum*, *t*-cinnamonaldehyde (Figure 1) along with its standard demonstrated the most significant antibacterial activities (*p* <0.05) against all bacterial strains except *E*. *coli* SS1. This is the only chemical entity identified from these essential oils which has illustrated growth inhibition activity against *P*. *fluorescens* (~15 mm) in bioautography assay. Moreover, *t*-cinnamonaldehyde has exhibited the highest growth inhibition zone (>31 mm) against *B*. *licheniformis* as well as the strains of *Salmonella spp*. Nevertheless, it failed to inhibit the growth of MDR *E*. *coli* SS1 strain.

The prime component of *A*. *subulatum* essential oil, eucalyptol (Figure 1) and its reference standard demonstrated good antibacterial activities against the strains of *Salmonella spp*. and *B*. *licheniformis*. This was followed by moderate growth inhibition of *E*. *coli*. However, eucalyptol as well as its standard did not inhibit the growth of *P*. *fluorescens* and *S*. *aureus* strains.

Similarly, in essential oil of *S*. *aromaticum*, eugenol (Figure 1) and its standard exhibited more or less similar antibacterial activities against strains from *Salmonella spp*. But in contrast to cuminaldehyde (*C*. *cyminum*’*s* oil), eugenol as well as its standard have strongly inhibited the growth of MDR *E*. *coli* SS1 strain with inhibition zone diameter of ~30 mm. These activities were clearly observed on TLC plates with big inhibition zone in case of *E*. *coli* and lack of any inhibition zone on TLC plates containing *P*. *fluorescens*.

In short, all of the major components of tested essential oils have showed very good antimicrobial activities against the strains of MDR *Salmonella spp*., while their activities against other bacterial strains are variable.

**Figure 2:**
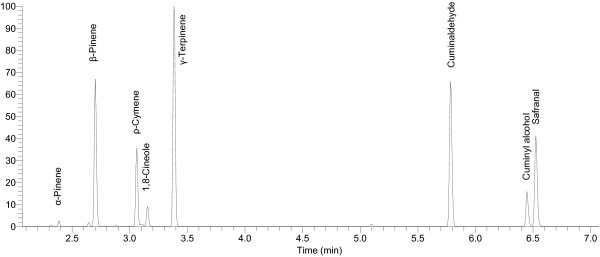
**Representative GC/MS chromatogram of essential oil from *Cuminum cyminum*.**

GC/MS analysis of essential oils showed that *C*. *cyminum* contained monoterpene hydrocarbons *γ*-terpinene (23.2%) and *β*-pinene (14.8%), followed by cuminaldehyde (17.2%) and safranal (10.8%) as a major components oxygenated monoterpenoids (Table 3). While minor components include cuminyl alcohol (3.9%) and 1,8-cineole (2.0%) as oxygenated monoterpenoids while *ρ*-cymene (7.6%) and *α*-pinene (0.5%) as monoterpenoid hydrocarbons (Figures 1 and 2). On the other hand, the most abundant components in the bark of *C*. *verum* essential oil were recognized as oxygenated monoterpenoids *t*-cinnamonaldehyde (4.3%) and eucalyptol (0.32%), along with minor components such as cuminaldehyde, *γ*-terpinene and an un-identified trace constituent. *A*. *subulatum* seed’s essential oil mainly consisted of oxygenated monoterpenoid eucalyptol (5.2%), whereas *γ*-terpinene, linalool, *β*-pinene and *α*-pinene were present in traces. The major components of *S*. *aromaticum* essential oil were identified as oxygenated monoterpenoids such as eucalyptol (4.6%) and eugenol (0.73%).

**Table 3:** Chemical composition, retention time and concentration (%) of essential oils by GC/MS analysis

**Essential oils**	**Compounds**	**Retention time**	**Concentration (%)**
*C. cyminum*	*α*-Pinene	2.38	0.5
	*β*-Pinene	2.70	14.8
	*ρ*-Cymene	3.06	7.6
	1,8-Cineole	3.15	2.0
	*γ*-Terpinene	3.38	23.2
	Cuminaldehyde	5.78	17.2
	Cuminyl alcohol	6.45	3.9
	Safranal	6.52	10.8
*C. verum*	Eucalyptol	3.14	0.32
	*γ*-Terpinene	3.37	Traces
	Cuminaldehyde	5.77	Traces
	*t*-Cinnamaldehyde	6.24	4.3
	Unidentified	7.79	--
*A. subulatum*	*α*-Pinene	2.38	Traces
	*β*-Pinene	2.70	Traces
	Linalool	3.10	Traces
	Eucalyptol	3.15	5.2
	*γ*-Terpinene	3.38	Traces
*S. aromaticum*	Eucalyptol	3.17	4.6
	Eugenol	7.52	0.73

## Discussion

Typhoid fever, a potentially fatal illness is caused by *S*. *typhi* and *S*. *paratyphi* Gram-negative bacteria. Towards the end of last century, these bacterial strains developed multidrug resistance to most of the available first-line treatment drugs (e.g. chloramphenicol, ampicillin, etc.) in various parts of the world including Pakistan [44]. This resistance shifted the focus of medical science to more effective classes of antibiotics, quinolones and cephalosporins for the treatment of typhoid. But unfortunately, resistance emerged gradually against these drugs as well [7-9,13,45]. Among tested bacteria, all clinical isolates including three strains of *Salmonella spp*., *E*. *coli* and *S*. *aureus* have been identified as multidrug resistant strains [7,37-40]. Essential oils are reported to posses, wide range of biological activities, especially these have been actively pursued as antimicrobial agents alone or in combination with other antibiotics. Notably, essential oils, when used in combination, have shown synergistic effects on the activity of antibiotics against which the microbes have already developed resistant. The essential oil of *C*. *cyminum* L decreased biofilm formation and enhanced the activity of the ciprofloxacin disk against *K. pneumonia*[46]. Similarly, this essential oil has improved the antimicrobial activity of nisin against food-borne pathogens [47].

The present study was conducted on essential oils of spices from Pakistani, which were extracted by hydro-distillation process. Different essential oils possessed significant (*p* <0.05) antimicrobial activity due to the presence of various bioactive compounds (Figure 1) [48]. Considering the MIC, *C*. *cyminum* essential oil was active against all tested bacterial species. It showed maximum activity against MDR *S*. *typhi* D1 Vi-positive and *E*. *coli* SS1 strains. This oil illustrated double activity (3.4 mg/ml) against *S*. *typhi* D1 Vi-positive (biofilm producing) strain than *S*. *typhi* G7 Vi-negative (biofilm non-producing) strain. It is generally believed that biofilm production is the characteristic which gives additional protection to the pathogenic microbes against host immune response and antibiotics [49,50]. On the basis of these facts, Vi-positive strain can be assumed more resistant from antimicrobials than Vi-negative strain. However, in this study *C*. *cyminum* essential oil gave higher activity against Vi-positive strain. This may be due to the fact that the surface chemistry of these two types of *Salmonella* strains may be somewhat different. Moreover, essential oil from *C. cyminum* is known to inhibit or decrease biofilm formation and has enhanced the activity of the ciprofloxacin disk against *Klebsiella pneumonia*[46]. This oil may have halted the proper assembly of cell membrane, which ultimately inhibited Vi-positive bacterial growth. Nevertheless, application of essential oil from *C. cyminum* resulted in cell elongation, repression of capsule expression and inhibition of urease activity in biofilm producing *K. pneumonia*[51]. So, the use of *C. cyminum* essential oil alone or with combination of the existing antibiotics, could really contribute in developing the strategy to deal with the MDR biofilm forming microbial pathogens.

*C. verum* essential oil exhibited excellent response against all bacterial species with best MIC values (Table 1), which are in accordance with previous literature [52]. Particularly, it has strongly inhibited the growth of *S. typhi* G7 (Vi-negative) and *P. fluorescens* strains. In contrast to this, lower activity of *C. cyminum* essential oil against genus *Pseudomonas* has been observed [53]. In MIC assay, *A. subulatum* essential oil was found to be effective against all tested bacteria at lower concentrations especially against MDR *E. coli* SSI strain. No previous biological activities of cardamom essential oil were reported against the currently selected bacterial species except *E. coli*[54] whose MIC value did not correlate with our findings, which may be due to the fact that different species of cardamom used in previous study. Similarly, MIC assay revealed good level of biological activities against all of the tested microorganisms. Moreover, all tested bacteria showed resistance against amoxicillin in MIC assay except *B. licheniformis* exhibiting MIC value as 0.02 ± 0.05 mg/ml (Table 1, see footnote).

TLC analysis showed that cuminaldehyde was present as a main component in *C. cyminum* essential oil, as the colored zone showed *R*_f_ value comparable with its reference standard. Presence of bands or zone of inhibition at same *R*_f_ value in the extract showed antimicrobial activity against various microorganisms [55]. Bioautographic system expressed the antibacterial activity by zone of inhibition against bacterial species tested except *P. fluorescens*. These results correlate with the previous studies [53] where the *C. cyminum* essential oil has given good activity against various plants and mushroom disease causing bacterial pathogens, but this oil in general indicated lower inhibition activities against bacteria belonging to the genus *Pseudomonas*. However, no previous reports were found related to TLC-bioautography of *C. cyminum* essential oil against the other tested microorganism species. TLC-bioautography of *C. verum* essential oil confirmed the antibacterial activity of *t*-cinnamaldehyde against tested bacterial species except *E. coli* (Table 2) which were in agreement with the findings of Horvath et al. [42]. TLC analysis confirmed the presence of eucalyptol as major constituent of *A. subulatum* essential oil by comparing with reference standard as colored zone by calculating *R*_f_ value. Bioautography showed that *A. subulatum* essential oil possessed antibacterial active agents by showing inhibition zone against all bacterial species except *S. aureus* and *P. fluorescens.* So far, no previous study is known related to TLC-bioautography of *A. subulatum* essential oil against the tested bacteria.

TLC-bioautography of *S. aromaticum* essential oil indicated the presence of bioactive agents by showing zone of inhibition against tested strains except *P. fluorescens*. These results were comparable to the previous findings [56]. TLC-bioautography of *S. aromaticum* essential oil against *S. aureus* revealed that lower concentration was effective in inhibiting tested bacteria which was more or less similar to the known literature [48]. Some of essential oils did not show inhibitory zones on TLC-bioautography against some bacterial strains, while they have showed activities in MIC assay in few cases. This might be due to the loading of less concentration of bioactive compound on TLC or evaporation of compounds and/or photo-oxidation [55].

Variation in the chemical profile of essential oils could influence their biological activities. Therefore, it was important to determine the chemical composition of essential oils to correlate with their antimicrobial activities [28]. Chemical profiling of essential oils were performed using GC/MS [15,43]. The analysis of *C. cyminum* essential oil indicated the presence of eight volatile components (Figure 2 and Table 3), characterized mainly by monoterpene hydrocarbons (*α*-pinene, *β*-pinene, *ρ*-cymene and *γ*-terpinene) and oxygenated monoterpene (1, 8-cineole, cuminaldehyde, cuminyl alcohol and safranal). Previous reports on chemical composition of *C. cyminum* essential oil showed that it had oil yield of 5.4% with major compounds cuminaldehyde, 1, 8-cineole, *ρ*-cymene, *γ*-terpinene and *β*-pinene which are almost similar to our results [36,57].

*t*-Cinnamaldehyde was identified as major component and eucalyptol as minor component in *C. verum* essential oil through GC/MS analysis (Table 3). These results were in accordance with previous reports [34] with some difference in concentration which could be due to seasonal variation, adaptive metabolism, parts of plant used, distillation process and other factors [58]. The GC-MS analysis of essential oil from *A. subulatum* (large cardamom) revealed oxygenated monoterpenoid eucalyptol as a major component and hydrocarbon monoterpene in traces (Table 3). While in literature volatile components of *Amomum cannicarpum* consisted of *β*-pinene, elemol and *α*-cadinol identified by GC/MS [35]. These results were found to be in contrast to our chemical profiling, which could be due to different sub-species comparison. The chemical composition of *S. aromaticum* essential oil showed the presence of oxygenated monoterpenes with eucalyptol as major component and eugenol in minor quantity. These results were not associated with previous studies [59], where chemical composition of *S. aromaticum* essential oil revealed eugenol, caryophyllyne and eugenyl acetate as major components. These variations in the chemical composition of essential oil might be due to ecological, climate and geographical conditions, time of harvesting and age of plant [60].

## Conclusions

On the basis of these results, it may be concluded that essential oils extracted from various Pakistani spices possess mixture of several volatile bioactive components, especially the oxygenated monoterpenoids showed strong antimicrobial activities against MDR and other bacterial strains. TLC-bioautography can be used to identify the bioactive components present in the essential oils. Essential oil of *C. verum* exhibited the excellent overall activities especially against MDR *S. typhi* Vi-positive, *S. typhi* Vi-negative, *S. paratyphi* A, *E. coli* and *S. aureus* strains with MIC values ranged from 2.9 to 4.8 mg/ml.

The tested essential oils can be pursued for therapeutic purposes as the potential antibacterial agents against MDR clinical and soil isolates because these bacteria are widely spread and are posing serious therapeutical problems due to their grave pathogenicities and high level of resistance. This is a unique study where the essential oils are fully analyzed and evaluated against various classes of MDR clinical and soil isolates.

Further studies will be required on these essential oils to explore their synergistic effects on the activities of the first line antibiotics against which these bacteria have already developed resistance. The identification of precise molecular mechanism addressing how these essential oils inhibit bacterial growth are need to be explored. Although essential oils known to have low toxicities per se, this cannot be assumed for individual components. Therefore, the toxicity studies of the active components will also be required.

## Competing interests

The authors declare that they have no financial and/or non-financial competing interests.

## Authors’ contributions

RN carried out experiments such as hydrodistillation, MIC, TLC-bioautography. IH participated in its design and coordination. AT conducted GC/MS analysis. MT contributed in setting MIC, TLC-bioautography. MR, SH & MSM helped in designing experiments & financially supported research. ABS analyzed the data by applying statistical tests. MI overall supervised research work and drafted the manuscript. All authors read and approved the final manuscript.

## Pre-publication history

The pre-publication history for this paper can be accessed here:

http://www.biomedcentral.com/1472-6882/13/265/prepub
